# Day-care, early common infections and childhood acute leukaemia: a multicentre French case–control study

**DOI:** 10.1038/sj.bjc.6600091

**Published:** 2002-04-08

**Authors:** F Perrillat, J Clavel, M F Auclerc, A Baruchel, G Leverger, B Nelken, N Philippe, G Schaison, D Sommelet, E Vilmer, D Hémon

**Affiliations:** Institut National de la Santé et de la Recherche Médicale, Inserm U170, 16 avenue Paul Vaillant Couturier, 94807 Villejuif, France; Institut National de la Santé et de la Recherche Médicale, Institute of Hematology, Saint-Louis Hospital, 75010 Paris, France; Department of Paediatric Haematology, Saint-Louis Hospital, 75010 Paris, France; Department of Paediatric Haematology, Armand Trousseau Hospital, 75012 Paris, France; Department of Paediatric Haematology-Oncology, Jeanne de Flandre Hospital, 59000 Lille, France; Department of Paediatric Oncology, Debrousse Hospital, 69009 Lyon, France; Department of Paediatric Oncology, Brabois Hospital, 54000 Nancy, France; Department of Paediatric Haematology-Immunology, Robert Debré Hospital, 75019 Paris, France

**Keywords:** childhood leukaemia, risk factors, day-care, early infections, breast-feeding

## Abstract

We conducted a case–control study to investigate the role of early infections in the aetiology of childhood acute leukaemias. The study included 280 incident cases (240 acute lymphoblastic leukaemia and 40 acute non-lymphoblastic leukaemia) and 288 hospital controls, frequency matched by age, gender, hospital, catchment area of the hospital and ethnic origin. Data were obtained from standardised face-to-face interviews of the mothers. The interviews included questions on early common infections, day-care attendance, breast-feeding, birth order and infantile diseases. Odds ratios were estimated using an unconditional regression model including the stratification variables, parental socio-economic status and perinatal characteristics. Birth order was not associated with childhood leukaemia (acute lymphoblastic or acute non-lymphoblastic). A statistically-significant inverse association was observed between childhood leukaemia and day-care attendance (odds ratio=0.6, 95% Confidence Interval=(0.4–1.0)), repeated early common infections (⩾4 per year before age two, odds ratio=0.6 (0.4–1.0)), surgical procedures for ear–nose–throat infections before age two (odds ratio=0.5 (0.2–1.0)) and prolonged breast-feeding (⩾6 months, odds ratio=0.5 (0.2–1.0)). In the multivariate model including day-care attendance, early common infections and breast-feeding, results concerning breast-feeding remained unchanged. A statistically significant interaction between day-care attendance and repeated early common infections was observed. When the interaction was taken into account, the simple effects of day-care and early common infections disappeared (odds ratio=1.1 (0.5–2.3) and odds ratio=0.8 (0.5–1.3), respectively) while the joint effect of day-care attendance and early common infections was negatively associated with childhood leukaemia (odds ratio=0.3 (0.1–0.8)). All the above associations were observed both for acute lymphoblastic leukaemia and acute non-lymphoblastic leukaemia. Our results support Greaves' hypothesis, even though they are not specific of common leukaemia.

*British Journal of Cancer* (2002) **86**, 1064–1069. DOI: 10.1038/sj/bjc/6600091 www.bjcancer.com

© 2002 Cancer Research UK

## 

Little is known about the aetiology of childhood acute leukaemia (AL), which is the most frequent childhood cancer world-wide (Doll, 1989; Ross *et al*, 1994). An infectious aetiology has been suggested for many years, particularly since specific viruses have been shown to be involved in leukaemia in animals (Essex, 1982). However, no specific virus has been found to explain childhood leukaemia. Kinlen postulated that childhood leukaemia occurs as a rare response to a specific infection(s) and increased by marked rural–urban population mixing (Kinlen, 1988, 1995; Kinlen *et al*, 1990; Kinlen and Petridou, 1995). Greaves hypothesised that common B-cell leukaemia, which is responsible for the incidence peak observed between ages 2 and 5 years, may result from a two-step process, with a first step occurring *in utero* (Greaves, 1988). Greaves suggested that the risk of childhood common B-cell leukaemia is increased by an immune proliferative stress. By contributing to the normal maturation of the immune system, early common infections or factors favouring infections in childhood would thus protect the child against leukaemia, while a situation of relative isolation would make the child more vulnerable (Greaves and Alexander, 1993; Greaves, 1997).

This article reports the results of a French case–control study designed to investigate the role of early common infections and factors influencing early common infections (day-care attendance, breast-feeding, and birth order) in childhood AL.

## SUBJECTS AND METHODS

### Subjects

A hospital-based case–control study was conducted in the hospitals of Lille, Lyon, Nancy and Paris (France). To be eligible, cases were required to be aged 15 years or less, reside in the hospital catchment area, and have a recent diagnosis of AL, i.e. diagnosis between January 1, 1995 and December 31, 1999. The hospital-based design of the study was chosen since case and control blood samples were required. Special care was therefore paid to selecting an appropriate control group. The controls were children hospitalised in the same hospital as the cases, mainly in orthopaedic and emergency departments, and residing in the catchment area of the hospital. Many different diagnostic categories were included in order to avoid selection biases in the event that a particular disease was related to the exposures of interest (Breslow and Day, 1980; Rothman and Greenland, 1998). However, children hospitalised for cancer or a major congenital malformation were not eligible for the study, since those diseases may share risk factors with leukaemia. Recruitment was frequency matched by age, gender, hospital, hospital catchment area and ethnic origin (Caucasian, North African, others). Of the mothers of the 282 cases and 291 controls who were eligible for interview during the interviewers' working hours, two cases and two control mothers refused to participate. We excluded one control child who was adopted. Thus, a total of 280 incident cases of AL confirmed by cytology, consisting of 240 cases of acute lymphoblastic leukaemia (ALL) and 40 cases of acute non-lymphoblastic leukaemia (ANLL), and 288 controls were included in the study.

### Data collection

The mothers of the cases and controls were interviewed when the index child was in complete remission or in good condition (on average, 2 months post-diagnosis), using a standard questionnaire administered by trained medical interviewers. Interviews were performed in the hospitals under strictly similar conditions for the cases and controls. Neither the parents nor the interviewers were informed of the hypothesis underlying the study. Data relating to early infections and factors promoting infections included: birth order of the index child; interval to birth of the immediately elder sibling (intervals less than 2 and less than 5 years were examined); duration of breast-feeding; history of day-care attendance; history of early common infections; history of surgical procedures for early ear–nose–throat (ENT) infections; and infantile diseases. ‘Repeated early common infections’ was defined as four or more common infections per year before age 2. Surgical procedures for early ENT infections were defined as: adenoidectomy, tonsillectomy, tympanostomy tube insertion and tympanocentesis before age 2 years. The procedures were used as a surrogate for early, repeated, ENT infections.

### Statistical analysis

All analyses were performed using the SAS computer software. Odds ratios (OR) were estimated using an unconditional logistic regression model including stratification variables, i.e. gender, age, ethnic origin and hospital. The socio-demographic characteristics (maternal educational level and parental socio-professional category) and perinatal characteristics (birth weight, length of pregnancy and number of pregnancies) were taken into account as potential confounders. The analyses of day-care attendance, early infections, breast-feeding and infantile diseases were conducted on the children aged over 2 years in order to be certain that early infections before age 2 would have already taken place in both the cases and controls. In the same way, multivariate analyses were conducted on the children aged over 2 years. Testing for interactions was systematically conducted. Two different final models were generated using two different variables as markers of early infections. In one model, ‘repeated infections before age 2’ and, in the other, ‘surgical procedures for ENT infections before age 2’ were used. In both, day-care and breast-feeding were included.

## RESULTS

Most of the controls (88%) were recruited in an orthopaedic or emergency department (Table 1

**Table 1:**
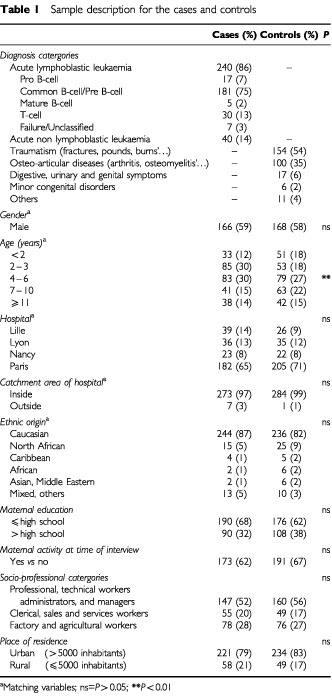
Sample description for the cases and controls

). Sixty per cent of the cases were 2–6 years old, *vs* 55% of the controls. The recruitment of controls in the age bracket 2–6 years (i.e. the childhood leukaemia incidence peak) was very difficult. Cases and controls were very similar with respect to gender, hospital, hospital catchment area, ethnic origin, maternal occupation at the time of interview, maternal educational level, parental socio-professional category and urban/rural residence status (Table 1). The cases and controls did not differ with respect to birth weight, length of pregnancy or number of pregnancies. However, reduced length of pregnancy and low birth weight were both, and independently, negatively related to prolonged breastfeeding (>6 months). Conversely, parity was positively related to prolonged breastfeeding.

No association between birth order and childhood leukaemia (ALL or ANLL) was observed (Table 2

**Table 2:**
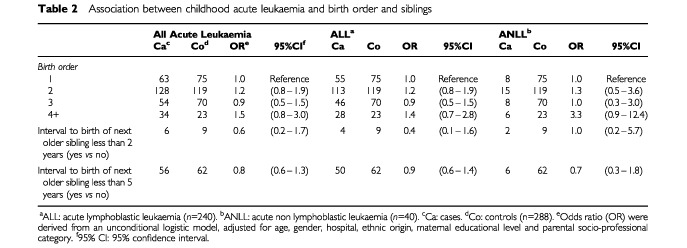
Association between childhood acute leukaemia and birth order and siblings

). The OR associated with a time interval to immediately elder sibling birth of less than 2 years was less than unity, but the association was far from statistical significance (OR=0.6, 95% Confidence Interval=(0.2–1.7)). The OR was close to unity when the interval to birth of the immediately elder sibling was less than 5 years (OR=0.8 (0.6–1.3)).

The results for early infections, day-care attendance and breast-feeding are shown in Table 3

**Table 3:**
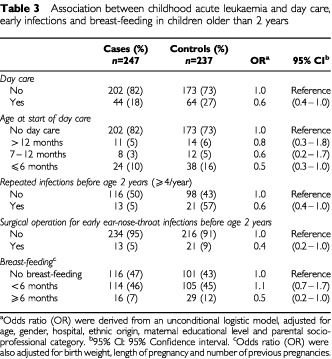
Association between childhood acute leukaemia and day care, early infections and breast-feeding in children older than 2 years

. A statistically-significant inverse association between day-care attendance and childhood AL (OR=0.6 (0.4–1.0)) was observed. The association was more pronounced for children having started day-care at age 6 months or less (OR=0.5 (0.3–1.0)) than for children having started day-care at age 13 months or more (OR=0.8 (0.3–1.8)). Nevertheless, the trend for age of starting day-care was not statistically significant. Repeated common infections before age 2 and surgical procedures for ENT infections before age 2 were statistically and negatively associated with childhood leukaemia (OR=0.6 (0.4–1.0) and OR=0.4 (0.2–1.0), respectively). Lastly, breast-feeding for at least 6 months was negatively associated with childhood leukaemia with an OR of 0.5 (0.3–1.1) and an OR of 0.5 (0.2–1.0) after adjustment for perinatal characteristics (birth weight, length of pregnancy, number of pregnancies).

The results of the joint analyses of early infection and breast-feeding are shown in Table 4

**Table 4:**
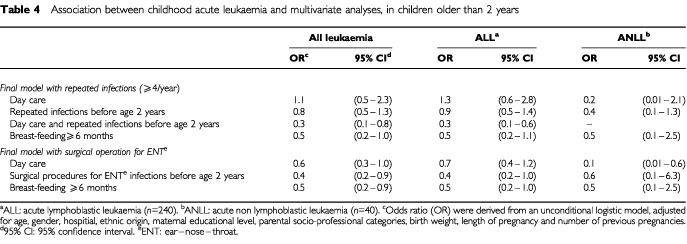
Association between childhood acute leukaemia and multivariate analyses, in children older than 2 years

. The model including day-care, repeated infections before age 2 and breast-feeding, showed a significant interaction between day-care attendance and common infections before age 2 (OR=0.3 (0.1–0.8)). The model including day-care, surgical procedures for ENT infections before age 2 and breast-feeding, did not show any interaction. For both models, the estimations were not altered by the mutual adjustments. Similar results were observed for ALL (common or not) and ANLL.

The variables of interest were identically distributed over the different diagnostic categories in the control group. Moreover, the estimations of the above associations remained the same when the control group was restricted to the main diagnostic categories, i.e. injury or osteoarticular diseases. The OR were: OR=0.3 (0.1–0.8) and OR=0.3 (0.1–0.7), respectively, for the joint effect of day-care and repeated early common infections; OR=0.4 (0.2–1.1) and OR=0.2 (0.1–0.8) for ENT infections before age 2; and OR=0.5 (0.2–1.3) and OR=0.4 (0.1–1.2) for breast-feeding.

No association was found between measles, rubella or chickenpox and childhood leukaemia (Table 5

**Table 5:**
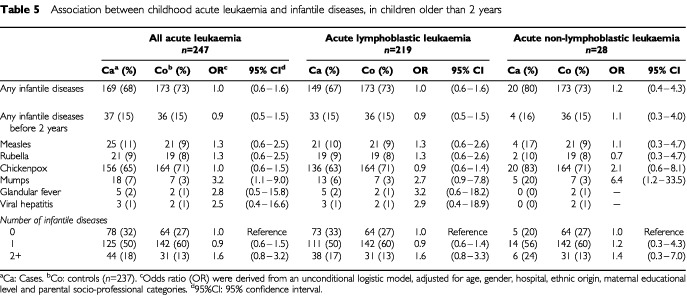
Association between childhood acute leukaemia and infantile diseases, in children older than 2 years

). Elevated OR were observed for the association between childhood leukaemia and glandular fever and viral hepatitis, but based on very small numbers. A significant elevated OR was associated with mumps (OR=3.2 (1.1–9.0)).

## DISCUSSION

Greaves suggested that early common infections in infancy and factors influencing early infections, such as birth order, older siblings, breast-feeding, and day-care, could have a protective effect against childhood AL (Greaves, 1988). A hospital-based case-control study was conducted in France to investigate the role of early infections in childhood AL. The hospital-based design of the study was chosen since case and control blood samples were required. Special care was therefore paid to selecting an appropriate control group. The reasons for which some case or control French-speaking mothers were not eligible for interview consisted in the non-availability or vacation of the interviewer, except for the mothers of two cases and two controls, who refused to participate. Controls were included from many diagnostic categories, none of those categories being related to the variables of interest. Our results were unchanged when the control group was restricted to each main diagnostic category. The cases and controls were very similar with respect to socio-demographic characteristics, i.e. maternal occupation at time of interview, maternal educational level, socio-professional categories and the rural/urban residential status.

Several previous studies on incident cases (Van Steensel-Moll *et al*, 1986; Petridou *et al*, 1997; Bener *et al*, 2001), and, in particular, several mortality studies (Stewart *et al*, 1958; MacMahon and Newill, 1962; Stark and Mantel, 1966) found that being the first-born increased the risk of, or mortality related to, childhood AL. We did not find such an association, in line with many other studies based on incident cases (Shaw *et al*, 1984; McKinney *et al*, 1987; Kaye *et al*, 1991; Savitz and Ananth, 1994; Cnattingius *et al*, 1995; Roman *et al*, 1996; Shu *et al*, 1999; Infante-Rivard *et al*, 2000; Neglia *et al*, 2000; Rosenbaum *et al*, 2000). An OR less than unity, but far from significance, was observed with respect to a time interval to birth of the immediately elder sibling of less than 2 years, as was reported by Kaye *et al* (1991), but not by Neglia *et al* (2000).

A statistically-significant inverse association was observed between day-care attendance and childhood AL, as has previously been reported by Petridou *et al* (1993) and Infante-Rivard *et al* (2000). That association was not observed in three other studies (Petridou *et al*, 1997; Neglia *et al*, 2000; Rosenbaum *et al*, 2000). It is noteworthy that, in Neglia's study (Neglia *et al*, 2000), children attended day-care more often than in our study (49% *vs* 27%), but started less often before age 1 than in our study (15% *vs* 21%). The statistically-significant interaction between day-care attendance and early common infections observed in our study suggests that infection in children attending day-care could differ in terms of frequency and/or type to those in other children. Diarrohea, upper respiratory tract infections and otitis have been shown to be more frequent in children attending day-care, compared to children not attending day-care (Haskins and Kotch, 1986; Wald *et al*, 1991; Reves *et al*, 1993). The statistically-significant inverse association between childhood AL and surgical procedures for ENT infection before age 2 is consistent with the results of a large study on ALL reported by Neglia *et al* (2000) in which the OR decreased as the number of episodes of otitis reported during the first year of life increases.

In our study, the surgical procedures for ENT infections before age 2 and day-care attendance among controls were significantly more frequent for urban residents than for rural residents. However, the cases and controls were similar with respect to urban/rural residential status, and our results remained unchanged when the analyses were restricted to urban children only.

Differential misclassifications such as under-declaration by the cases' mothers and/or over-declaration by the controls' mothers would seem minimal in the present study, due to the fact that the same standardised conditions were used to interview both the cases and the controls. Moreover, we obtained consistent results with respect to the mothers' declarations of their child's common infections before age 2 and the history of ENT surgery before age 2. The latter constitutes a less sensitive but more specific and more readily remembered surrogate of early infections. Similar results regarding the risk of childhood AL and early infections have already been reported in other studies. A negative association with infections during the first year of life was observed by Van Steensel-Moll *et al* (1986). McKinney *et al* (1999) observed a negative association with neonatal infections. Our results are also consistent with those of Neglia *et al* (2000). In contrast, two studies found no association with early infection (McKinney *et al*, 1987; Dockerty *et al*, 1999).

Breast-feeding for at least 6 months was statistically-significantly and negatively associated with childhood AL. That finding has also been reported in several recent case-control studies (Schüz *et al*, 1999; Shu *et al*, 1999; Smulevich *et al*, 1999; Infante-Rivard *et al*, 2000; Bener *et al*, 2001). Two studies found a reduced risk of childhood leukaemia, although the reductions were not significant (Davis *et al*, 1988; Dockerty *et al*, 1999). Other studies did not, however, evidence any association (Van Steensel-Moll *et al*, 1986; Magnani *et al*, 1988; McKinney *et al*, 1987; Golding *et al*, 1990; Shu *et al*, 1995; Petridou *et al*, 1997; Rosenbaum *et al*, 2000; Hardell and Dreifaldt, 2001). Except for two studies, one conducted in Shanghai (Shu *et al*, 1995) and the other in Sweden (Hardell and Dreifaldt, 2001), the duration of breast-feeding was not considered (Van Steensel-Moll *et al*, 1986; McKinney *et al*, 1987; Magnani *et al*, 1988; Golding *et al*, 1990; Petridou *et al*, 1997; Rosenbaum *et al*, 2000).

The usual infantile diseases – chickenpox, rubella and measles – were not associated with childhood AL. That finding is consistent with the results of recent studies (Dockerty *et al*, 1999; Schüz *et al*, 1999). McKinney *et al* (1987) observed an elevated OR (OR=4.1 (1.5–11.3)) between viral diseases comprising chickenpox, rubella, measles, mumps, viral meningitis, viral influenza and the risk of childhood leukaemia and lymphoma. In our study, an elevated and significant OR was also found for mumps (OR=3.2 (1.1–9.0)).

In conclusion, the main findings of the present study were the inverse relationships between childhood AL and early common infections, day-care and prolonged breast-feeding. These results are consistent with other publications and support Greaves' hypothesis, even though they are not specific to ALL.
